# Supplementary material for: Nuclear envelope-dependent heterochromatin positioning gates the timing of nucleolar assembly in the early embryo
Source: bioRxiv. 2026 Jul 22:2026.07.20.739428. Preprint. [Version 1] doi: 10.64898/2026.07.20.739428 (PMC13419420; doi:10.64898/2026.07.20.739428)
Supplement: Supplementary file 1 [file NIHPP2026.07.20.739428V1-supplement-1.pdf]

bioRxiv preprint doi: https://doi.org/10.64898/2026.07.20.739428; this version posted July 22, 2026. The copyright holder for this preprint (which was not certified by peer review) is the author/funder, who has granted bioRxiv a license to display the preprint in perpetuity. It is made available under a CC-BY 4.0 International license.

SUPPLEMENTAL FIGURES

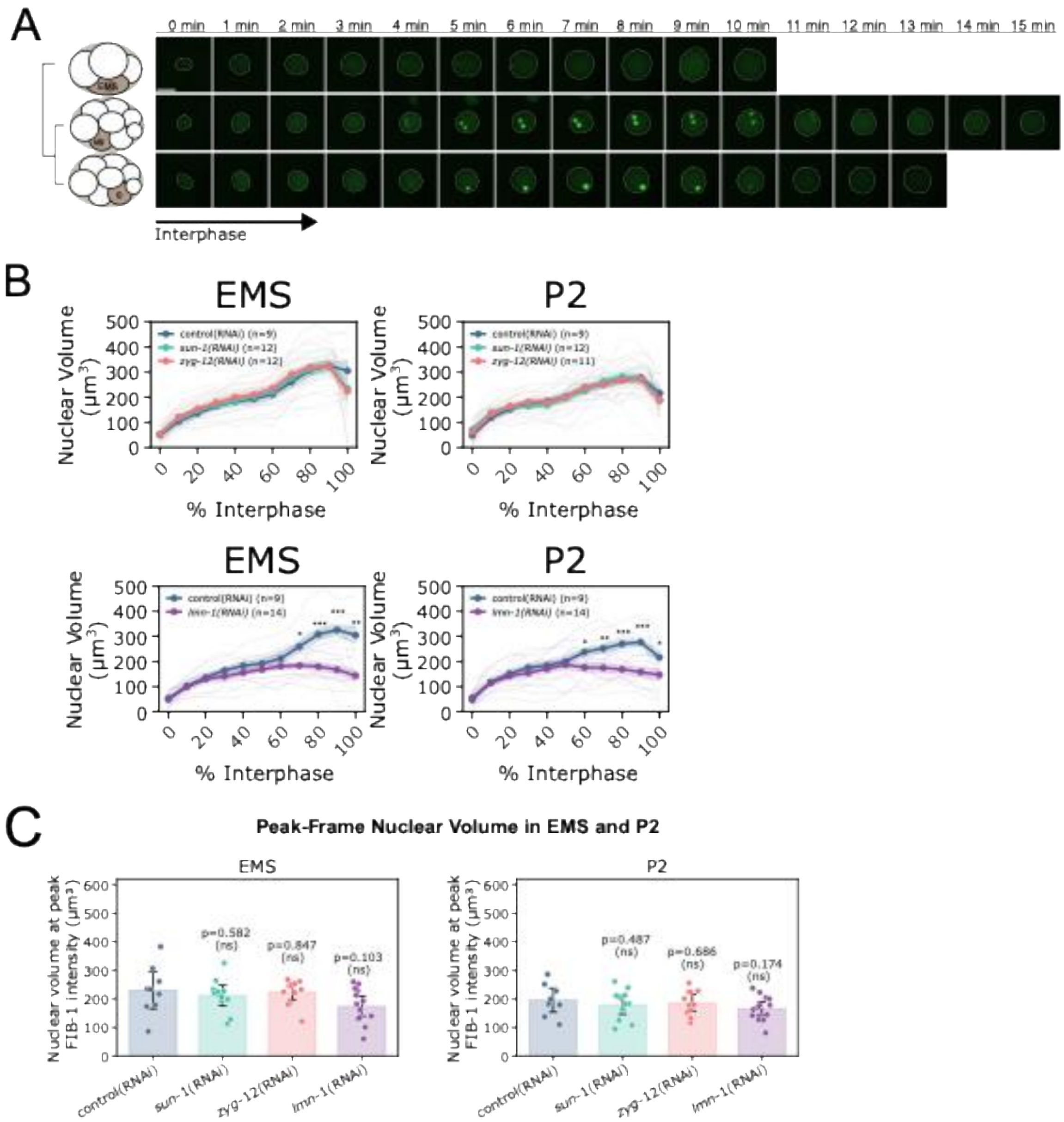

Figure S1. Nuclear volume changes do not account for precocious nucleolar assembly in *sun-1(RNAi)* or *zyg-12(RNAi)* embryos. (A) Representative time-course images of FIB-1 signal in the EMS, MS, and E nuclei during interphase. Images are maximum-intensity projections; dashed outlines indicate the segmented nuclear boundary. Time is shown in minutes relative to the start of the displayed interphase interval. All images are displayed with the same green LUT and identical intensity range for comparison. Scale bar, 5 µm. **(B)** Nuclear volume trajectories across normalized interphase in EMS and P2 nuclei after control(RNAi), *sun-1(RNAi)*, *zyg-12(RNAi)*, or *lmn-1(RNAi)*. Lines indicate mean; shading indicates SEM. Asterisks mark significant differences from control(RNAi) at matched time points by two-sided Welch's t-test; *p <

bioRxiv preprint doi: https://doi.org/10.64898/2026.07.20.739428; this version posted July 22, 2026. The copyright holder for this preprint (which was not certified by peer review) is the author/funder, who has granted bioRxiv a license to display the preprint in perpetuity. It is made available under a CC-BY 4.0 International license.

0.05, **p < 0.01, ***p < 0.001. n values are indicated in each plot. **(C)** Nuclear volume at the frame of peak FIB-1 intensity in EMS and P2 nuclei. Each point represents one nucleus; bars indicate mean ± 95% CI. Peak frame was defined as the frame with maximal FIB-1 intensity for each nucleus. p values compare each RNAi condition with control(RNAi) by Welch's t-test; ns, not significant. n values for control(RNAi)/*sun-1(RNAi)/zyg-12(RNAi)/lmn-1(RNAi)*, respectively, are: EMS, 9/12/12/14; P2, 9/12/11/14. n indicates the number of nuclei analyzed for each blastomere and condition.

bioRxiv preprint doi: https://doi.org/10.64898/2026.07.20.739428; this version posted July 22, 2026. The copyright holder for this preprint (which was not certified by peer review) is the author/funder, who has granted bioRxiv a license to display the preprint in perpetuity. It is made available under a CC-BY 4.0 International license.

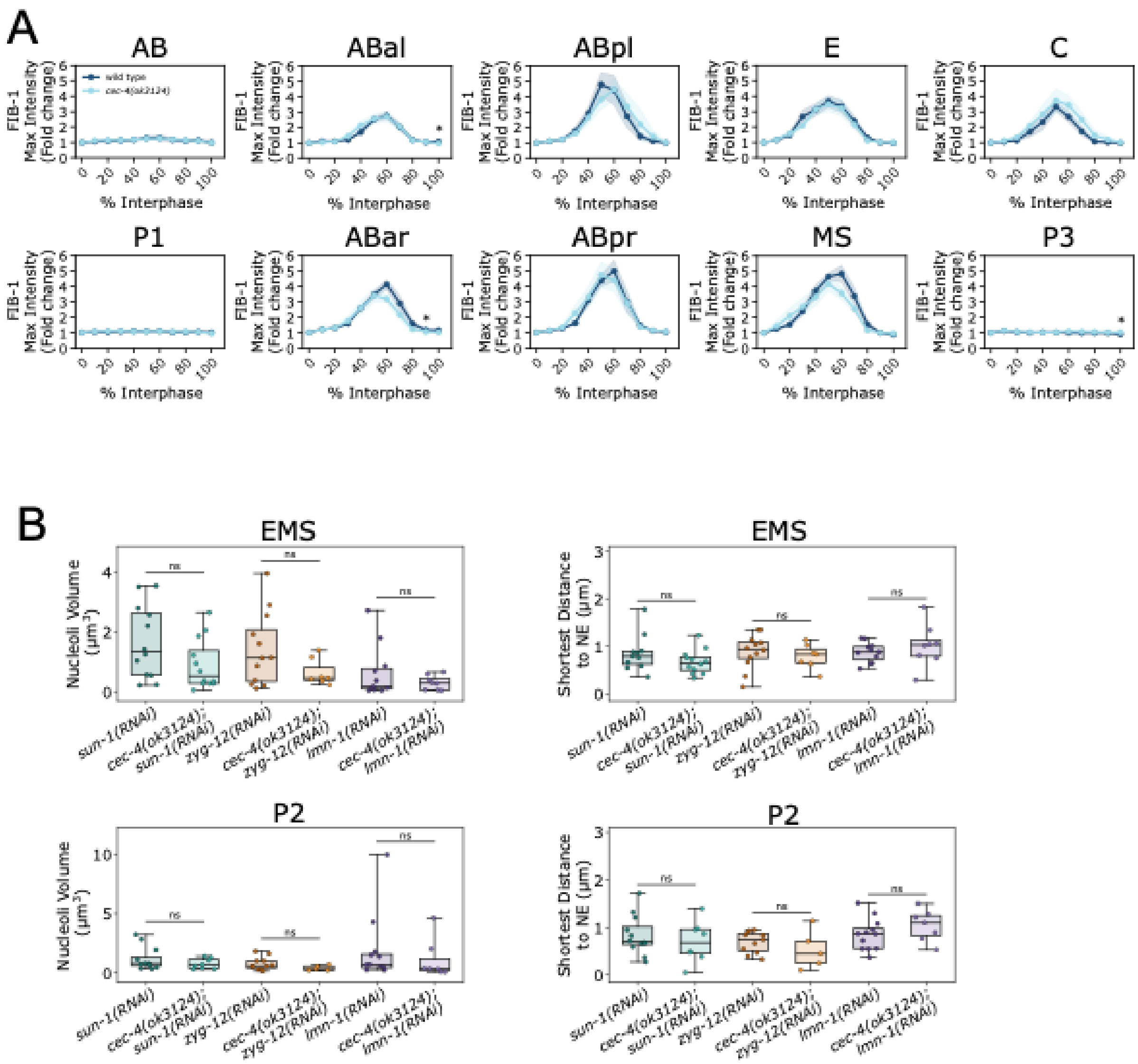

Figure S2. CEC-4 loss has limited effects on 8-cell-stage nucleologenesis and does not alter nucleolar size or position in double-perturbed embryos. (A) Normalized maximum FIB-1::eGFP intensity traces for AB, P1, and 8-cell-stage lineages in wild-type and *cec-4(ok3124)* embryos. FIB-1 intensity was normalized to the first tracked frame and plotted across normalized interphase. Lines indicate mean; shading indicates SEM. Asterisks mark significant differences between wild type and *cec-4(ok3124)* at matched time points by two-sided Welch's t-test; *p < 0.05. n values for wild type/*cec-4(ok3124)*, respectively, are: AB, 3/5; P1, 5/6; ABal, 8/11; ABar, 5/6; ABpl, 4/5; ABpr, 3/6; MS, 8/10; E, 8/11; C, 4/6; P3, 7/5. n indicates the number of nuclei analyzed for each blastomere and condition. **(B)** Single-nucleolus measurements in EMS and P2 nuclei from *sun-1(RNAi)*, *cec-4(ok3124)*; *sun-1(RNAi)*, *zyg-12(RNAi)*, *cec-4(ok3124)*; *zyg-12(RNAi)*, *lmn-1(RNAi)*, and *cec-4(ok3124)*; *lmn-1(RNAi)* embryos. Panels show nucleolar volume and absolute shortest distance to the nuclear envelope measured in 3D. Each point

bioRxiv preprint doi: https://doi.org/10.64898/2026.07.20.739428; this version posted July 22, 2026. The copyright holder for this preprint (which was not certified by peer review) is the author/funder, who has granted bioRxiv a license to display the preprint in perpetuity. It is made available under a CC-BY 4.0 International license.

represents one embryo/file mean, calculated from all nucleoli measured within that embryo for the indicated lineage. Boxes indicate the median and middle 50% of embryo/file means, with whiskers showing the full range. Statistical comparisons between matched single and double perturbations were performed on per-embryo means; ns, not significant.

 3

bioRxiv preprint doi: https://doi.org/10.64898/2026.07.20.739428; this version posted July 22, 2026. The copyright holder for this preprint (which was not certified by peer review) is the author/funder, who has granted bioRxiv a license to display the preprint in perpetuity. It is made available under a CC-BY 4.0 International license.

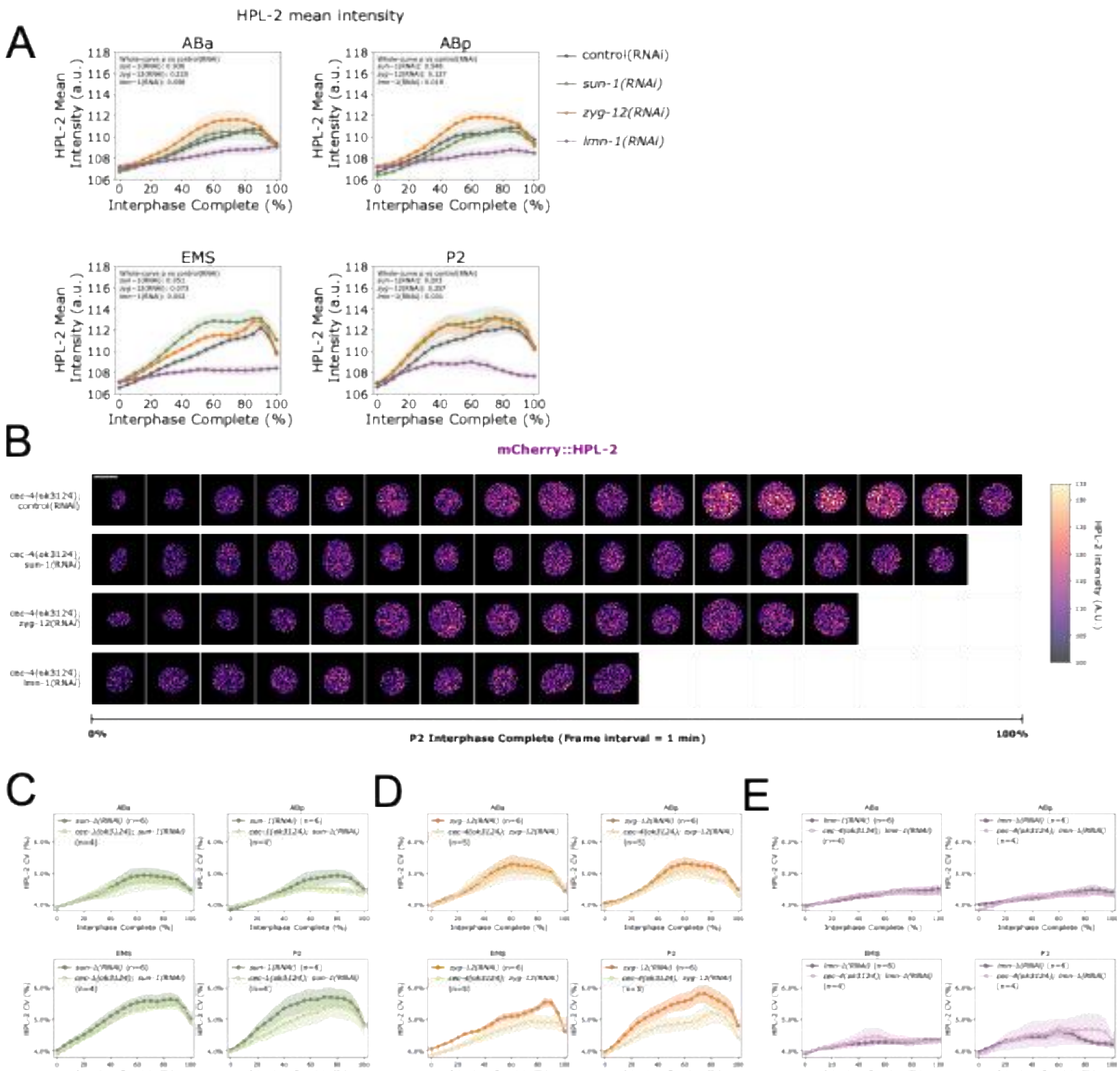

Figure S3. HPL-2 intensity levels and spatial heterogeneity during interphase in single- and double-perturbed embryos. (A) HPL-2 mean fluorescence intensity across normalized interphase in ABa, ABp, EMS, and P2 nuclei in control(RNAi), *sun-1(RNAi)*, *zyg-12(RNAi)*, and *lmn-1(RNAi)* embryos. Lines show mean; shading indicates SEM. p values (two-sided Welch's t-test on whole-curve AUC/100) compare each condition with control(RNAi). **(B)** Representative time series of mCherry::HPL-2 in P2 nuclei during normalized interphase in cec-4(ok3124) embryos with the indicated RNAi conditions. At each displayed time point, the single z-plane containing the maximum FIB-1 signal within the tracked P2 mask was selected independently, and the corresponding mCherry::HPL-2 signal from that same plane is shown. Frame interval, 1 min. Scale bar, 5 µm. **(C-E)** HPL-2 CV in 3D across normalized interphase in ABa, ABp, EMS, and P2 nuclei for each double-perturbed condition compared with its corresponding single RNAi. Lines indicate

bioRxiv preprint doi: https://doi.org/10.64898/2026.07.20.739428; this version posted July 22, 2026. The copyright holder for this preprint (which was not certified by peer review) is the author/funder, who has granted bioRxiv a license to display the preprint in perpetuity. It is made available under a CC-BY 4.0 International license.

means; shading indicates SEM. p values: two-sided Welch's t-test on whole-curve AUC/100.

bioRxiv preprint doi: https://doi.org/10.64898/2026.07.20.739428; this version posted July 22, 2026. The copyright holder for this preprint (which was not certified by peer review) is the author/funder, who has granted bioRxiv a license to display the preprint in perpetuity. It is made available under a CC-BY 4.0 International license.

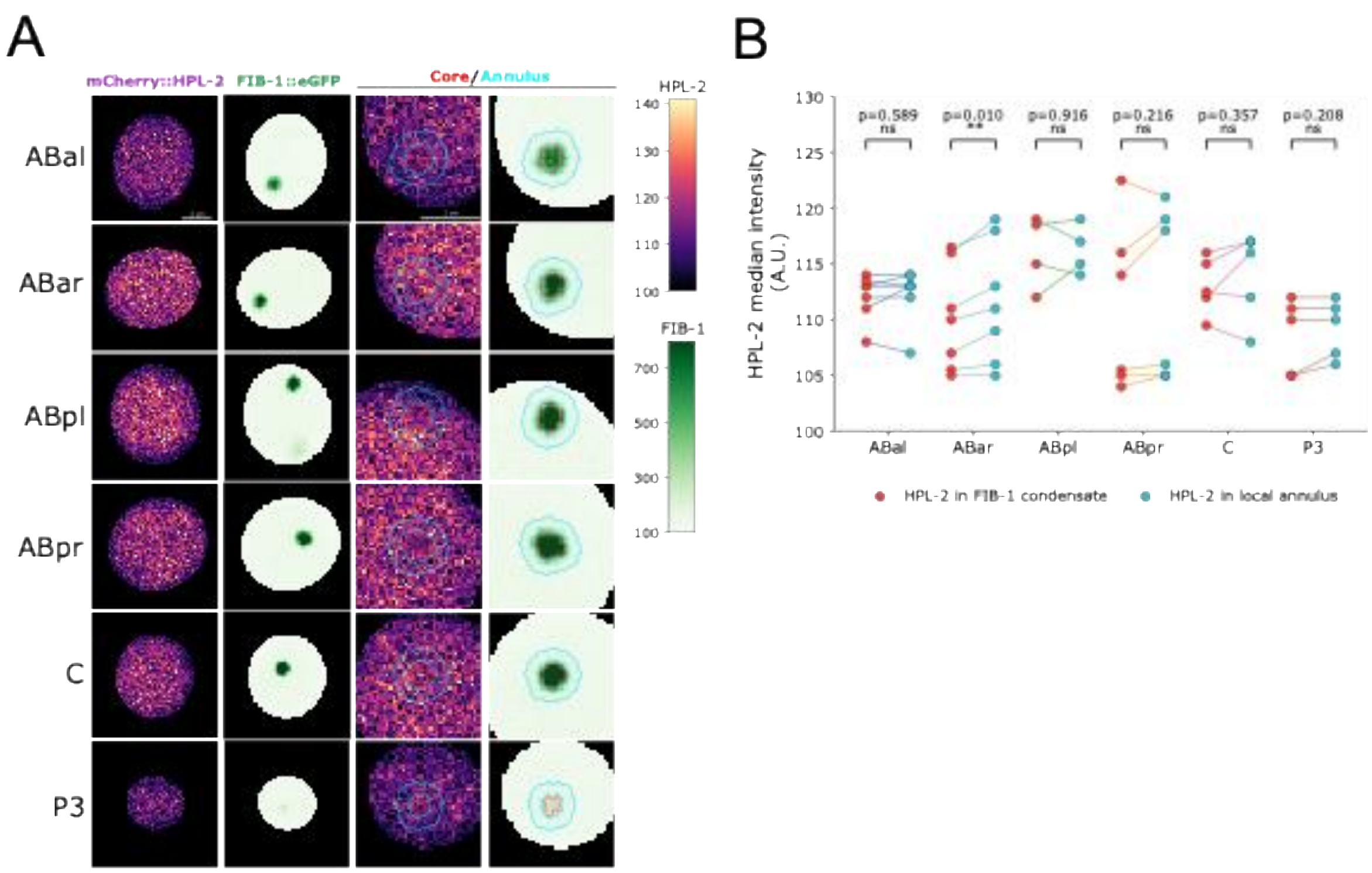

Figure S4. Local HPL-2 signal at FIB-1 condensates in additional 8-cell-stage lineages. (A) Representative single-plane mCherry::HPL-2 and FIB-1::eGFP images of wild-type ABal, ABar, ABpl, ABpr, C, and P3 nuclei at the peak FIB-1 frame. Left to right: full HPL-2 plane, corresponding FIB-1 plane, magnified HPL-2 image, and magnified FIB-1 image. Red contours indicate FIB-1 condensate cores; cyan contours indicate the corresponding local annuli. Scale bars, 2 µm. **(B)** Paired median HPL-2 intensity within each FIB-1 condensate core (red points) and its local annulus (cyan points). One condensate per embryo was selected based on the strongest FIB-1 core-to-annulus enrichment; HPL-2 signal was not used for selection. Connected points indicate matched regions from the same nucleus. Exact unadjusted p values were calculated using two-sided paired t-tests and are shown above each lineage; **p < 0.01; ns, not significant. n = 7, 7, 4, 6, 5, and 5 independent embryos for ABal, ABar, ABpl, ABpr, C, and P3, respectively. Corresponding analyses of E and MS are shown in Figs. 5C-D.

Movie S1. Depleting LINC complexes SUN-1 or ZYG-12 advances nucleolar assembly to 4-cell stage. Representative time-lapse confocal maximum-intensity projections of FIB-1::eGFP in embryos treated with control(RNAi), *sun-1(RNAi)*, or *zyg-12(RNAi)*. Movies are shown side by side with matched display settings. Time is shown in minutes. The movie pauses at 15 min to highlight precocious, distinct FIB-1 condensates in the EMS and P2 blastomeres at the 4-cell stage in *sun-1(RNAi)* and *zyg-12(RNAi)* embryos, compared with the absence of distinct nucleolar assembly in control(RNAi). Scale bar, 10 µm.

bioRxiv preprint doi: https://doi.org/10.64898/2026.07.20.739428; this version posted July 22, 2026. The copyright holder for this preprint (which was not certified by peer review) is the author/funder, who has granted bioRxiv a license to display the preprint in perpetuity. It is made available under a CC-BY 4.0 International license.

Movie S2. LMN-1 depletion promotes persistent FIB-1 condensate growth in EMS and P2. Representative time-lapse confocal maximum-intensity projections of FIB-1::eGFP in embryos treated with control(RNAi) or *lmn-1(RNAi)*. Movies are shown side by side with matched display settings. Time is shown in minutes. The movie pauses at 15 min in control(RNAi) and at 17 min in *lmn-1(RNAi)* to indicate the EMS and P2 blastomeres. At this stage, transient, indistinct FIB-1 condensates in control(RNAi) have already dissolved, whereas FIB-1 condensates in *lmn-1(RNAi)* continue to grow into distinct condensates and persist. Scale bar, 10 μm.
